# Austin District Medical Society

**Published:** 1888-04

**Authors:** T. D. Wooten, T. J. Bennett

**Affiliations:** President; Secretary


### Society Notes.

### AUSTIN DISTRICT MEDICAL SOCIETY.

[President, Dr. T. D. Wooten ; Secretary, Dr. T. J. Bennett.]

The third regular quarterly meeting of this Society was held in Austin, March 8, 1888, with a good attendance.

The following physicians were admitted to membership—thus swelling the roll to over fifty active members—to-wit: Drs. H. H. Thorpe, Liberty Hill ; J. A. Holloway and S. E. Hudson, of Round Rock ; Jno. Preston, 1st Assistant Physician, State Lunatic Asylum, Austin ; Frank M. D. Hill, of Dunlap.

Dr. R. S. Gregg, of Manor, read a paper on the "Treatment of Abortion." (This paper appears in this issue of the Journal.)

Dr. A. N Denton, who had been appointed to open discussion, said, he was much pleased with the paper. The subject is as extensive as it is important—and while Dr. Gregg's paper was a very excellent one—and of practical value, it could not be expected in so short a space to cover the whole ground.

No subject in practice had given him as much trouble and anxiety as abortion, and he did not know of any definite rule or plan of treatment that could be relied on in all cases. The treatment must necessarily depend upon the stage in which we find the case. Dr. Gregg spoke of the use of opiates. If we are not sure that sufficient damage has been done; if the placenta is not sufficiently detached to destroy the vitality of the ovum, opium is useful; we surely should prevent abortion if possible, but in endeavoring to prevent it where it is already impossible to prevent it, we may do much harm, and there are cases even, where the administration of opium may be indicated, and may do good in relieving pain, even after detachment ; but there is danger of producing inertia—thus favoring hemorrhage, and rendering it difficult to void the contents of the womb—and thus, by the retention of some part of the decidua, septic troubles may result and a long train of evils follow, even after you are sure the ovum has escaped. There is a prevalent idea that if abortion occurs once, it is more apt to do so again; that a habit may be established. His experience led him to believe that it is more apt to occur at the third or fourth month; and at that time is very hard to manage. On the whole he thinks Dr. Gregg has indicated about the proper course and treatment in dealing with abortions.

As to prevention, where it is thought unusually apt to occur, some years ago "black haw" was much in vogue, and was thought to possess almost specific properties. He had a succession of cases and gave it a thorough trial, and was disappointed. Rest in bed, and hips elevated, as suggested in Dr. Gregg's paper, are the safest means; if the patient has suffered abortion before, he would insist on putting her to bed by all means.

As to after-treatment, he had nothing to add to Dr. Gregg's suggestions; cleanliness, antiseptics, etc. The sequellæ are sometimes serious; one of the causes of fibroids of the walls, is the placenta torn loose from its implantation.

On the whole he commends Dr. Gregg's paper. Thinks it is a very good one and possessed of practical value.

Dr. Q. C. Smith said:

"To say nothing of the immediate or remote *cause* of abortions, but merely to refer briefly to the operative treatment of an abortion already in active process, and hence, as a rule, its consummation inevitable, we would say: Supposing the hemorrhage more or less free, and other signs and symptoms usually accompanying this condition, to exist, we would place the patient in Sims' position and with a Sims' speculum, or some modification thereof, bring to view the os uteri; and if it be found closed and rigid, dilate it well with a metalic cervical dilator, and proceed at once to remove the entire product of conception. To do this, the finger may be sufficient, and should be tried first; but no time should be wasted in prolonged vain digital efforts, as the dull curette, or placental forceps, or polypus forceps may be used, if careful, skillful manipulation be exercised, with perfect safety, to clean out the uterine cavity. Then staunch the hemorrhage at once, first, by washing out the uterus with dilute hot iodine water, or hot dilute boric acid solution, injected through a tube of large returning capacity, watching the os uteri, all the while closely observing the progress of events. The hemorrhage having ceased entirely, now mop the entire endometrium with Churchill's tinct. iodine. The patient should now be made confortable, and treated as the general constitutional indications of the case may demand. Tinct. cinnamon, ipecac, and quinine, are favorite constitutional hemostatics with us, in this class of cases; ergot is not a reliable constitutional hemostatic in the early months of pregnancy, and may be given—teaspoonful fl. ext. t. d.—with impunity regularly, and with benefit to patients, in the earlier months of pregnancy, when such patients suffer from hemoptysis, as we know from repeated experience."

Dr. W. S. Richmond, First Vice President, had had good results from the black-haw in preventing abortion where it was thought the habit existed, giving it regularly for sometime previous to the expected attack. Had not used it as a means of arresting a threatened abortion.

Dr. L. H. Hardy, Second Vice President, said that the doctors as a rule are pretty well up in the treatment of abortion ; but it requires nice judgement to determine just where to cease efforts to prevent, and to conduct the case to a thorough and complete evacuation. The removal of the decidua is a matter of vital importance, and yet there is no definite and positive means of knowing when it has all escaped ; and yet we must know. Had a case which came near terminating fatally, because he took the word of the patient and attendants that "everything had come away." At the next visit, fever, and searching for himself found secundines imprisoned, and had much difficulty in getting it away. Thinks well of black-haw as a preventive.

Dr. B. E. Hadra had not heard the paper, and did not know what had had been said as to habitual abortion. The ohly way to prevent habitual abortion is to subject both husband and wife to a course of anti-syphilitic treatment, as seventy per cent of the cases were due to syphilitic contamination, and they occur usually at third or fourth, though he has known them to happen as late as the sixth and seventh month. Of course, there are exceptions. As a rule, he avacuates the womb as rapidly as possible, if danger is threatening ; else, leave nature to take her course. Few know how difficult it is to get into the womb, with the tedious means of dilatation generally at hand. Is opposed to tamponing ; it is possible to thus conceal a dangerons hemorrhage. Prefers Barnes' dilator; but there is a little invention, a century old, perhaps, which has been lately revived, that he resorts to most generally where dilatation is necessary and difficult, i. e. to incise the os; nicking, or one incision will often make the os yield. This is the best and shortest way; then, after a few minutes we can enter the womb; and the best and safest way is to remove the secundines with the fingers. As to preventing abortion, he has no confidence in remedies, and has tried all. If any have value it is ergot in small quantities. Ergot gives tonicity to the womb. Thirty to forty drops of ergot injected into the rectum, is better than any quantity by the mouth; seems so to him, at any rate; but his experience has not been large enough, perhaps, to base any opinion on.

Dr. T. J. Tyner said he would like to call attention to one point in the etiology of abortion. Sometimes a fatty degeneration of the placenta is found. Remembered a confrere, whose wife had suffered a number of abortions, and on examination, the placenta was found enlarged and full of fat globules. When this lady became again pregnant, she was put upon chlorate potassa, which was at one time regarded as a specific, and she went on to full term.

Dr. T. J. Bennett said: "Whatever may be the primary cause or causes of abortion, whether syphilis, fatty degeneration of the placenta, mal-positions of the uterus, or cervical rents, to my mind, the exciting cause is hemorrhage. It is poured out between the decidua and uterine wall, and by the stimulus of distention labor is brought on. The tampon is a fine agent to bring about this condition. It prevents the flow of blood through the cervix, especially if saturated with alum water and placed firmly against the os. This plan should be adopted where abortion is inevitable, hemorrhage not alarming and pain inadequate. If the patient is worn and needs sleep, I administer an opiate and expect, after a rest of a few hours, to have no further trouble.

"The best uterine dilator is the finger, but when the hemorrhage is threatening and a quicker method demanded, the Goodell dilator is indispensible."

Dr. Hadra would like to set himself right—he had been misunderstood. He would not do away with the tampon altogether,—only when there is a dangerous hemorrhage, he wouldn't use it. He would give ergot when he found a flabby womb, and want of contraction; not otherwise.

Dr. W. A. Ellison said the profession was much divided as to the value of the viburnum prunifolium, as a prophylactic in habitual abortion. He had had fine effects from it, better than from opiates. Morphine will stop pain, but it also arrests the secretions, which viburnum does not. He often combines viburnum, ergot and laudanum, and finds it better than either drug used singly. Viburnum, in teaspoonful doses, will often arrest a threatened abortion.

Dr. Manney had a case of threatening abortion in which he gave everything he could think of, in the vain endeavor to prevent it, and finally concluded it was unavoidable, and that he would hasten it; accordingly he gave ergot, and to his surprise everything was instantly quieted. Had had no good from the use of viburnum.

Dr. Q. C. Smith demonstrates his use of instruments in inevitable abortion.

Dr. Wm. S. Wallace, representing Mariani & Co., New York, was present with samples of Vin Mariani Coca Erythroxylin—which he gave to the physicians present.

Dr. Wooten, the president, being called upon for his views as to the subject under discussion said:

The paper which Dr. Gregg had favored the society with, was on the treatment of abortion,—but the discussion had taken the range of the entire subject, etiology, pathology' treatment, etc In his judgment, with regard to abortion—in the majority of cases—a cause can be discovered. and he has generally found that cause to be either reflex, or due to mal positions of the uterus—some of the forms of flexions or versions. He doubts the existence of habitual abortion without some definite and tangible cause. Often we may arrive at, combat, or remove the influences favoring this mishap. Lacerations, rectal troubles, and even gonorrhoæ being amongs them. Fatty degeneration of placenta as an excitant of abortion is a sequel of syphilis. He recalled the case of lady in Kentucky who had at the time he visited her, had five premature deliveries from this cause, and almost at full term. The last time he attended her she had had nine; examined placenta and found it so softened that a stream of water would waste it away. He determined to bring on labor as rapidly as possible, in the effort to save the child, but it did not live—although the lady was near full period. This case was an exception to what I have said, and not due to syphilis.

As to the treatment, he has no confidence in the means usually resorted to. The viburnum had given him very litlle satisfaction. If the attachments are sufficiently disconnected abortion will ensue. If there is much loss of blood it will most likely take place; but not necessarily, if there is slight hemorrhage from the os. If you find abortion inevitable and the hemorrhage alarming, nothing can be better than the prompt removal of the entire contents of the womb. To do this it is often necessary to forcibly dilate the os, and in his judgment this is the best measure, and the fingers the best means. When there is sufficient dilatation, expulsion will take place. Or if the fingers cannot be used, carry up a dilator, and if the effort of dilatation is not too rapid the os will give way. He does not regard the curette as an intelligent means of removal. By manipulation, by pressing on fundus and using the finger and thumb as dilators, he can, in the majority of cases, effect a clean removal of uterine contents. This process he regards as safer than Barnes' or Goodell's or any other instrument, or tents, and insures a clean removal; thus all septic trouble is avoided.

Ergot had not effected much in his hands; especially until dilatation has been effected or has occurred. It is only in the later stages of gestation that ergot has a decided influence on the muscular fibres: he doubts its efficacy in the early stages. When the ovum is engaged in the os, a dose of ergot may expel it.

Dr. Gregg's paper was very satisfactory; he regarded it as a very able paper, and especially commendable for its practical value, considering its brevity; it is *multum in parvo*.

Dr. A. N. Denton said he must differ with Dr. Hadra as to syphilis being responsible for 70 per cent. of the abortions; such was not in accordance with his observation, experience or reading.

Dr. Hadra: "I said habitual abortions."

Dr. Denton: "I beg pardon."

As to the value of the incision spoken of by Dr. Hadra, he never saw a case in which he thought it necessary or proper to incise the os. Rapid dilatation is the best means; as to leaving the case to nature after death of the fœtus he differs with the Doctor.

In such cases the fœtus is a foreign body, and a great risk of septic trouble. It should not be allowed to remain after its vitality has ceased. By ergot, or by mechanical means he would produce uterine contractions and expulsion of contents.

DR. GREGG would like to ask Dr. Wooten when we should cease our efforts to prevent an abortion?

DR. WOOTEN: "When there is distinct evidence of extinct vitality of the ovum; my judgment is, if hemorrhage continues, and you are satisfied the life of the ovum is destroyed you should remove it and all appendages forthwith."

The paper was referred to the Publishing Committee, and by them elected for publication in the Society's organ—DANIEL'S TEXAS MEDICAL JOURNAL."

The Society voted thanks to Dr. Gregg for his paper.

Adjourned till 2 o'clock, p. m.

---

AFTERNOON SESSION.—2 O'CLOCK.

Vice President W. T. RICHMOND in the chair.

DR. A. N. DENTON, late Superintendent State Lunatic Asylum, read a paper, entitled:

"THE MANAGEMENT AND TREATMENT OF THE INSANE IN TEXAS."

[This paper appears in this issue of the JOURNAL.]

Dr. Q. C. Smith thought it proper that some expression from the society should be had on this subjct. It is a matter of burning shame and disgrace to the medical profession of Texas that matters referred to by Dr. Denton should exist without solemn protest from them. Look at other States: no such condition exists. Before growing indignant at recital of wrongs and mismanagement in other States, we should look at home and institute reform.

DR. PRESTON, first assistant physician at the asylum said: It seems there is an impression abroad that the patients at the State Lunatic Asylum are in bad condition; was sorry that physicians have not more generally visited the asylum and seen for themselves. He is not superintendent, but has much to do with the management and hygiene of the establishment: and begs that before the visiting members of this association retµrn to their homes they will come out to the asylum and see for themselves, that the condition is not so bad as it has been represented. Agreed with Dr. Denton, that it is bad policy to change superintendent and other officers every two years, and thinks when competent men are found for the places they should be retained. Dr. Denton started out on the management of the asylum, but went into a history of the institution, with which I am familiar.

Dr. Daniel said that as Dr. Preston seemed to feel that Dr. Denton's remarks reflected somewhat on him, as to the condition of the asylum, he felt it to be only just to Drs. Dorsett and Preston, to bear testimony to the present good sanitary conditions of the institution. He had recently been shown through every department of the asylum, and confessed that he was both surprised and gratified to find everything scrupulously clean and home-like; and the inmates apparently happy and contended, seeming pleased to see Dr. Dorsett, when he visited the wards: surprised, he said, because of the numerous reports recently in circulation as to mismanagement.

Dr. Q. C. Smith believed that the present set of officers do their duty; would not be understood as condemning them; but he did unequivocally condemn the policy of the government in regard to changing the medical officers.

Dr. Denton had made no war on the institution, nor upon the officers; and therefore had no apology to make; believed that every word he had uttered, was true. His object had been to show the bad effects of frequent changes in the administration of these institutions,—especially with reference to the medical officers; He had tried to present the truth; would say, however, that to a visitor, the *appearance* of the wards, etc., is no indication as to the internal management of the institution. His object in treating of the subject before this association was to endeavor to get the medical profession of Texas, to urge the establishment af a better and permanent system of administration of the medical offices of these institutions.

Dr. Preston asked Dr. Denton if the appearance presented by the internal condition of the asylum is no indication of the efficiency of the management, what is?

Dr. Denton said that to fully answer the question would open up a discussion which it was unnecessary to go into.

Dr. Bennet said, in pursuance of Dr. Denton's object—as stated above— he would move that this Society wants the endorsement of the organized medical profession, or, rather, their co-operation, in the endeavor to procure a law on this subject, making the tenure of office of medical superintendent contingent on satisfactory administration, and

Dr. Denton then moved the appointment of a committee to take the matter in hand. This was amended by Dr. Bennett, who proposed the delegates from the Austin District Medical Society to the State Medical Association be instructed to bring the subject before that body, and to ask the co-operation of the profession in securing such law.

Dr. Daniel said, the object sought by Drs. Bennett, Denton and others, of getting this importnnt subject properly before the profession, could be best attained, and the sense of this Society best expressed, in a resolution which he had drawn up, as follows—and moved it as a substitute for previovs motions :

*Resolved,* That it is the sense of the Austin District Medical Society, that the law, with reference to the appointment of a Medical Superintendent for the State Lunatic Asylum and other eleemosynary institutions of the State is defective, and needs amending; and that the policy of frequent changes in those officers is detrimental to the welfare of the inmates ;

*Resolved,* That the delegates from the Austin District Medical Society to the Texas State Medical Association be instructed to bring up the subject at the coming meeting at Galveston, and endeavor to secure some action from that body looking to a change of the law.

Adopted.

Dr. L. W. Cocke, of San Marcus, read a very spicy and interesting paper, entitled:

" Is the World Satisfied with the Medical Profession ?"

[This paper, together with a synopsis of the discussion which followed, will appear in next issue. Ed.] .

Dr. Wooten, the President entering the room, Dr. Richmond, Vice-President, yielded the chair to him.

[Dr. Richmond related a case of placenta prævia successfully treated, which gave rise to a most spirited and interesting discussion, which we will endeavor to report in full in the May number of the JOURNAL; being obliged to omit it here for want of room. Ed.]

NEW BUSINESS.

DR. WOOTEN, from committee on securing a room in the new capitol, reported that he had almost positive assurance of a room for the exclusive use of the medical profession of the State, a place for our county and our district societies to meet, and—why not the State Medical Association? The library of the latter could be there, at least, and it would not involve, necessarily, "locating the association." The American Medical Association has movable meetings, and has its library at Washington too. Should the secretary happen to be a resident of Austin—which is not at all necessary however, the archives of the association could be kept in this room, and visiting physicians would always have a place of congeniality and interest to visit; could make it a headquarters during their stay. To procure such a room may require an act of the legislature, still those in charge seem to think there will be no difficulty in our finally securing a good room. It may be even on the second floor, but almost certainly on the third floor.

DR. BENNETT wished to memorialize the State Medical Association, urging them to put in an application for such room—for library and museum,—but Dr. Wooten said that his recollection was that Drs. Swearingen, McLaughlin and himself had been appointed a committee by the State Medical Association years ago, for this purpose, and now that the time had arrived for action,—the capitol completed,—he thought it would be in order for the committee to report at the coming meeting of the State Association.

The subject of delegates to the convention of the State Medical Association coming up, Dr. Richmond moved that a committee be appointed to select delegates; it was desirable to appoint only those who would attend, and it would be necessary to canvass the members to ascertain this important point. Carried.

The chair appointed Drs. Richmond, Bennett and Daniel to select delegates. [We regret that we are unable to announce the delegates here, the selection has not yet been made. The District Society, with fifty members, is entitled to ten delegates.—ED.]

The time and place of next meeting will be announced later; Austin, no doudt, some time in June. The Association is rapidly growing, and the meetings are well attended, and full of interest.

